# Air Pollution and Acute Respiratory Response in a Panel of Asthmatic Children along the U.S.–Mexico Border

**DOI:** 10.1289/ehp.1003169

**Published:** 2011-09-06

**Authors:** Stefanie Ebelt Sarnat, Amit U. Raysoni, Wen-Whai Li, Fernando Holguin, Brent A. Johnson, Silvia Flores Luevano, Jose Humberto Garcia, Jeremy A. Sarnat

**Affiliations:** 1 Department of Environmental Health, Rollins School of Public Health, Emory University, Atlanta, Georgia, USA; 2 Department of Civil Engineering, University of Texas at El Paso, El Paso, Texas, USA; 3 Division of Pulmonary, Allergy, and Critical Care Medicine, University of Pittsburgh Medical Center, Pittsburgh, Pennsylvania, USA; 4 Department of Biostatistics, Rollins School of Public Health, Emory University, Atlanta, Georgia, USA; 5 Interdisciplinary Health Science Ph.D. Program, College of Health Sciences, The University of Texas, El Paso, TX, USA; 6 Centro de Investigación y Desarrollo Tecnológico, Tecnológico de Monterrey, Campus Ciudad Juárez, Ciudad Juárez, Chihuahua, Mexico

**Keywords:** air pollution, asthma, children, exposure assessment, fine particulate matter, nitrogen dioxide, ozone

## Abstract

Background: Concerns regarding the health impact of urban air pollution on asthmatic children are pronounced along the U.S.–Mexico border because of rapid population growth near busy border highways and roads.

Objectives: We conducted the first binational study of the impacts of air pollution on asthmatic children in Ciudad Juarez, Mexico, and El Paso, Texas, USA, and compared different exposure metrics to assess acute respiratory response.

Methods: We recruited 58 asthmatic children from two schools in Ciudad Juarez and two schools in El Paso. A marker of airway inflammation [exhaled nitric oxide (eNO)], respiratory symptom surveys, and pollutant measurements (indoor and outdoor 48-hr size-fractionated particulate matter, 48-hr black carbon, and 96-hr nitrogen dioxide) were collected at each school for 16 weeks. We examined associations between the pollutants and respiratory response using generalized linear mixed models.

Results: We observed small but consistent associations between eNO and numerous pollutant metrics, with estimated increases in eNO ranging from 1% to 3% per interquartile range increase in pollutant concentrations. Effect estimates from models using school-based concentrations were generally stronger than corresponding estimates based on concentrations from ambient air monitors. Both traffic-related and non–traffic-related particles were typically more robust predictors of eNO than was nitrogen dioxide, for which associations were highly sensitive to model specification. Associations differed significantly across the four school-based cohorts, consistent with heterogeneity in pollutant concentrations and cohort characteristics. Models examining respiratory symptoms were consistent with the null.

Conclusions: The results indicate adverse effects of air pollution on the subclinical respiratory health of asthmatic children in this region and provide preliminary support for the use of air pollution monitors close to schools to track exposure and potential health risk in this population.

The impact of urban air pollution on asthmatic children has been a long-standing environmental health concern. For the U.S.–Mexico border region, this concern is pronounced because of rapid population growth near busy border highways and roads. Collectively, the total population living along the U.S.–Mexico border is approximately 12 million, a figure expected to double by 2030 (Peach and Williams 2004). Commensurate with this demographic change has been an increase in binational commerce along the border since the implementation of the North American Free Trade Agreement. Together, these trends have led to increases in the total number of international border crossings of gasoline and diesel motor vehicles, traffic congestion, and corresponding idling times at the crossings (Currey et al. 2005). The Paso del Norte (PdN) region, encompassing the metropolitan areas of Ciudad Juarez, Mexico (CJ), and El Paso, Texas, USA (EP), warrants specific attention, being one of the two busiest border crossing regions along the entire U.S.–Mexico border (Bureau of Transportation Statistics 2009), with 14–17 million annual vehicle border crossings at five separate locations.

Previous studies conducted in the PdN region report elevated traffic-related and non–traffic-related pollutant concentrations (e.g., Li et al. 2001). A recent critical review of the literature found sufficient evidence to support a causal relationship between traffic-related air pollution exposure and asthma exacerbation, particularly in asthmatic children (Health Effects Institute 2010). Results from an earlier study in CJ were consistent with this evidence base, with associations observed between road and traffic densities and airway inflammation in asthmatic children (Holguin et al. 2007). Pollutants not generally associated with traffic exposures, such as particulate matter (PM) with aerodynamic diameter ≤ 10 μm (PM_10_), have also been associated with pediatric asthma exacerbations in this region (Hernandez-Cadena et al. 2000), which may reflect effects of other health-relevant local sources, including wind-blown or road dust. Despite these findings, reliable indicators of air pollution exposures and accurate estimates of the associated health burden are difficult to obtain for the PdN region. For example, it is unlikely that the current local ambient monitoring network (three sites in CJ, eight sites in EP) adequately captures spatiotemporal trends in traffic-related emissions that exist (Gonzales et al. 2005), particularly because many sites do not measure relevant traffic-related pollutants.

To address these gaps, we conducted the first binational health effects study of air pollution on a panel of asthmatic children in CJ and EP, with a focus on traffic-related exposures. Spatially and temporally resolved monitoring of specific PM species and nitrogen dioxide (NO_2_) was conducted inside and outside of four schools in the study area, with concurrent measures of respiratory health collected on each child. Here, we assess *a*) whether school-based monitors are more effective than ambient monitors for research and guiding policy, *b*) which measures of traffic pollution are the most sensitive predictors of changes in the children’s health, and *c*) whether differences in risk are observed across different population subsets.

## Methods

**Figure 1:**
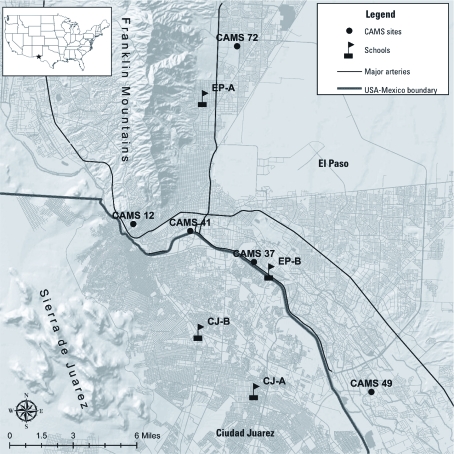
Map of study area, including location of air quality monitoring sites and schools.

*Study overview.* We conducted a repeated measures panel study of asthmatic children from two schools in CJ and two schools in EP. Schools were selected to maximize the gradient of potential traffic-related air pollution concentrations. In each city, we recruited one school located in a light traffic zone (CJ-A and EP-A; each surrounded by residential two-lane roads) and a second school in a heavy traffic zone (CJ-B and EP-B; each located within 300 feet of principal arterials, or high-service capacity controlled-access roadways, with heavy truck traffic; CJ-B was also located adjacent to the largest bus station in CJ) (Figure 1).

Four 15-child school-based “cohorts” were targeted for recruitment with the following study inclusion criteria: age between 6 and 12 years, self-reported physician’s diagnosis of asthma, current asthma medication use or asthma-related respiratory symptoms in the preceding year, and living in a home with nonsmokers. Health outcome and pollutant measurements were collected at each school for 16 consecutive weeks from January through May 2008. The study protocol was approved and overseen by the Institutional Review Board at Emory University. Each child’s legal guardian provided informed consent, children 11–12 years of age provided written assent, and children 6–10 years of age provided verbal assent to participate in the study; all forms were available in both English and Spanish, translated by a native speaker. Participants were provided with contact information of both native English- and Spanish-speaking study investigators in case of questions. Children received a $50 gift certificate to a local store for their participation.

*Health outcome data collection.* Health sampling sessions took place each Friday throughout the study period. A fraction of the sampling sessions (15% of all measurements) were conducted on Thursdays because of scheduling conflicts, such as school holidays. Exhaled nitric oxide (eNO), a sensitive and noninvasive biomarker of airway inflammation, was measured for each child during each sampling session using a NIOX MINO^®^ airway inflammation monitor (Aerocrine AB, Solna, Sweden) (Khalili et al. 2007) following the standard American Thoracic Society recommendations for eNO sampling (American Thoracic Society 2006). Two acceptable measurements were collected for each participant at each sampling session. Estimates of absolute precision (root mean squared difference/2^1/2^; mean = 2.0 ppb) and relative precision (100% × absolute precision/grand mean of measurements; mean = 8.8%) were obtained from a comparison of the paired duplicate samples by subject (Hoek et al. 2002); overall relative precision was within our target range of < 10%. The mean of each pair of duplicate samples was used in epidemiologic analyses. Each week, the children’s guardians completed daily diaries that provided information on the incidence of respiratory symptoms, school absenteeism, and medication use.

*Air quality data collection.* Concurrent indoor and outdoor measurements of PM and NO_2_ were collected at each school over the study period. Coarse PM (PM_10–2.5_; ≤ 2.5–10 µm), PM_2.5_ (≤ 2.5 µm), and black carbon [BC; a surrogate of elemental carbon (Cyrys et al. 2003)] were collected during two 48-hr (Monday–Wednesday, Wednesday–Friday) sampling sessions each week, and NO_2_ was collected over one 96-hr (Monday–Friday) sampling session. Logistical considerations precluded our ability to collect more temporally resolved NO_2_ measurements. Samplers were deployed at each school between 0830 hours and 1130 hours on start days, such that pollutant sampling end times coincided with the start of health sampling sessions at each school. For the 15% of health measurements conducted on Thursdays, air sampling was adapted accordingly when possible; however, the air sampling period of Wednesday–Friday remained for 11.7% of these days. Outdoor measurements were conducted on the roofs of each school. Indoor measurements were conducted in a room where the children were likely to spend time and that was considered to be broadly representative of the specific in-school microenvironment; across the four schools, indoor monitoring locations were a computer room, a library reference room, a classroom, and a library.

A complete description of the sampling methods and protocol can be found elsewhere (Raysoni et al. 2011). Briefly, PM_10–2.5_ and PM_2.5_ mass was collected using Harvard cascade impactors (Demokritou et al. 2002) and quantified via gravimetric analysis. PM_10_ levels were calculated as the sum of PM_10–2.5_ and PM_2.5_. BC was determined via particle reflectance measurements of the PM_2.5_ filters; absorption coefficients (per meter × 10^–5^) were converted to BC mass concentrations (micrograms per cubic meter) assuming a conversion factor of 1 (Wolfson M, Liu Y, Harvard School of Public Health, personal communication). Correlations among BC and absorbance values were therefore equal to 1 by definition. Given the variability of this coefficient (Cyrys et al. 2003), the absolute BC levels reported should be viewed cautiously. The choice of this nontemporally varying conversion factor is unlikely to have introduced analytical bias in our health effects models, which examined temporal associations between the pollutant metrics and the corresponding health endpoints. NO_2_ was collected using passive badge samplers and extracted and quantified via ion chromatography at the Harvard School of Public Health (Boston, MA).

Air quality data, which comprised hourly PM_10_, PM_2.5_, NO_2_, ozone (O_3_), temperature, and relative humidity, were collected from five continuous air monitoring stations (CAMS) in EP, operated by the Texas Commission on Environmental Quality (Figure 1). Hourly values were aggregated to 24-, 48-, 72-, and 96-hr averages, ending on Fridays to match the school-based measurements. CAMS 41 was chosen *a priori* as the primary monitoring site of interest because of its central location in the study area and availability of all air quality parameters of interest.

*Epidemiologic analysis.* We examined longitudinal associations between the various air pollution exposure metrics and respiratory response using generalized linear mixed models. For models predicting eNO, we used linear mixed effect models (PROC MIXED in SAS, version 9.2; SAS Institute Inc., Cary, NC) with pollutants modeled as fixed effects and subjects modeled as random effects. Subject-specific eNO variance increased with the mean, indicating that log-transforming the eNO values was appropriate. We included additional control for the repeated nature of the outcome data using a first-order autoregressive heterogeneous covariance structure. Generalized estimating equations with a first-order autoregressive correlation matrix produced similar effect estimates as the linear mixed-effects models in sensitivity analyses. We used logistic models (PROC GENMOD in SAS, version 9.2) with a first-order autoregressive covariance structure to predict the occurrence of the respiratory symptoms cough, wheeze, difficulty breathing, missed school, and short-acting bronchodilator (SABA) use.

As *a priori* covariates in all models, we controlled for school, ambient temperature, and relative humidity. Averaging times for the meteorologic variables were matched to the corresponding air pollutant variables of interest. Even though our eNO measurement method was designed to remove nitric oxide (NO) from inhaled air, in models predicting eNO we included control for indoor NO levels based on previous research supporting this approach (Dorevitch et al. 2007). Indoor NO levels were significant predictors of eNO, and associations between eNO and pollutant metrics were attenuated by 12% on average without indoor NO in the models; however, excluding indoor NO did not affect the overall significance of associations. Subject-specific factors age, sex, race, body mass index (BMI), hay fever status, cold symptoms, and inhaled corticosteroid (ICS) and leukotriene blocker (LT) use were also considered as potential covariates. Inclusion of these factors did not change the interpretation of our results and were thus omitted from the main models. These factors were considered as effect modifiers in secondary analyses using product terms with air pollution. We also conducted sensitivity analyses including season as a potential confounder; the conclusions from these models agreed with those of our reported results.

We compared associations *a*) among the various exposure metrics, *b*) among the different pollutants, and *c*) among the four school-based cohorts. Comparison of exposure metrics included ambient (CAMS 41) data and outdoor school and indoor school data (i.e., matching each subject to their respective school-specific measurements). To assess the relative contribution of pollutant metric versus subject-specific factors in our analyses, for each eNO–pollutant association we computed cohort-specific results (obtained from product terms of school and pollutant metric in the models); this allowed for simultaneous comparisons of effects among the pollutants for each cohort and effects among the cohorts for each pollutant.

To make valid comparisons, exposure metrics were matched for missing values. For example, if data for pollutant A were missing from school A for a specific date, data for all pollutants from all schools were set to missing for that date. We selected *a priori* exposure windows of the previous 48 hr (i.e., Wednesday–Friday) for PM species and 96 hr (Monday–Friday) for NO_2_. We conducted further analyses to examine the sensitivity of our results to exclusion of environmental tobacco smoke (ETS)–exposed subjects, the specification of lag structure, meteorologic control, and potential confounding by copollutants. Effect estimates for the log-transformed eNO measurements are presented as the percent change in eNO per increase in pollutant concentration (Fischer et al. 2002; Holguin et al. 2007; Liu et al. 2009). To compare the magnitude of effect across different metrics (i.e., ambient, outdoor, indoor) of the same pollutant, we scaled effects to interquartile range (IQR) increases in pollutant concentrations determined from the distribution of all measurements for overall analyses or from measurements at each school for cohort-specific analyses.

## Results

*Study population.* Fifty-eight asthmatic children completed the study protocol, with 14 or 15 children participating from each school. Children lived near their schools, with average home-to-school distances of < 2 miles. Except for one CJ-B subject residing closer to CJ-A than to CJ-B, the four cohorts did not overlap geographically. The overall mean age of the children was 8.7 (range, 6–12) years of age (Table 1). Age and sex distributions were similar across the cities and school-based cohorts, but significant differences were observed for other subject characteristics. Mean BMI for age and sex percentiles (Centers for Disease Control and Prevention 2011) were significantly higher among children in CJ than among those in EP, ranging from 50.6 (EP-A) to 83.9 (CJ-A). A significantly greater number of children experienced hay fever or seasonal allergies in EP (55%) than in CJ (28%). Similarly, a significantly greater number of children used ICS medication in EP (34%) than in CJ (3%). The number of children whose caretakers had less than a high school education was significantly greater in CJ (63%) than in EP (17%), which may indicate overall lower socioeconomic conditions for CJ than for EP children and corresponds to observations made by field staff. School lunch program participation, which is based on income eligibility in the United States, may also provide an indication of socioeconomic conditions in the EP schools. School lunch rates suggested lower socioeconomic conditions at EP-B, where 99% of children receive free lunch, than at EP-A, where 53% of children receive free lunch. There is no comparable school lunch program in CJ schools.

**Table 1:** Study population characteristics and outcomes summary.

All subjects	City					School								
	Characteristic	CJ	EP	*p*-Value*^a^*	CJ-A	CJ-B	EP-A	EP-B	*p*-Value*^a^*
*n*		58		29		29				14		15		15		14		
Age, years [mean (range)]		8.7 (6–12)		8.6 (6–12)		8.8 (6–12)		0.592		8.2 (6–12)		8.9 (6–12)		8.6 (6–10)		9.1 (6–12)		0.552
Sex [*n* (%) male]		38 (66)		17 (59)		21 (72)		0.269		7 (50)		10 (67)		10 (67)		11 (79)		0.464
Race [*n* (%)]																		
Black		4 (7)		0 (0)		4 (14)		0.010		0 (0)		0 (0)		4 (27)		0 (0)		< 0.001
Hispanic		51 (88)		29 (100)		22 (76)				14 (100)		15 (100)		8 (53)		14 (100)		
White		3 (5)		0 (0)		3 (10)				0 (0)		0 (0)		3 (20)		0 (0)		
BMI, lb/in^2^ [mean (range)]*b*		19.2 (13.7–35.0)		19.5 (14.6–27.3)		18.8 (13.7–35.0)		0.592		19.9 (15.8–27.3)		19.2 (14.6–26.7)		18.0 (15.0–35.0)		19.8 (13.7–31.3)		0.682
BMI percentile [mean (range)]*b*^,c^		64.6 (8.1–99.7)		74.4 (17.5–99.5)		55.6 (8.1–99.7)		0.019		83.9 (63.2–99.3)		66.8 (17.5–99.5)		50.6 (11.3–99.7)		61.0 (8.1–99.0)		0.034
BMI category [*n* (%)]*b*^,c^																		
Normal		35 (63)		15 (56)		20 (69)		0.409		5 (42)		10 (67)		12 (80)		8 (57)		0.243
Overweight		9 (16)		6 (22)		3 (10)				5 (42)		1 (6)		1 (7)		2 (29)		
Obese		12 (21)		6 (22)		6 (21)				2 (16)		4 (27)		2 (13)		4 (14)		
Hay fever [*n* (%)]		24 (41)		8 (28)		16 (55)		0.033		4 (29)		4 (27)		8 (53)		8 (57)		0.221
Medication use [*n* (%)]																		
ICS		11 (19)		1 (3)		10 (34)		0.005		1 (7)		0 (0)		7 (47)		3 (21)		0.004
ICS + LT		8 (14)		1 (3)		7 (24)		0.026		1 (7)		0 (0)		5 (33)		2 (14)		0.045
Caretaker education < high school [*n* (%)]*b*		21 (42)		17 (63)		4 (17)		0.002		9 (69)		8 (57)		2 (20)		2 (15)		0.012
eNO																		
*nd*		787		371		416				170		201		216		200		
Median (range), ppb		20.0 (2.5–135.0)		18.5 (2.5–125.0)		23.0 (18.5–135.0)		< 0.001		14.0 (2.5–85.0)		22.0 (5.0–125.0)		21.3 (2.5–119.5)		26.0 (3.8–135.0)		< 0.001
eNO in non-ETS-exposed subjects																		
*nd* (no. of subjects)		637 (47)		310 (24)		327 (23)				148 (12)		162 (12)		184 (13)		143 (10)		
Median (range), ppb		20.0 (2.5–135.0)		17.5 (2.5–109.0)		28.0 (2.5–135.0)		< 0.001		12.5 (2.5–85.0)		21.0 (5.0–109.0)		25.8 (2.5–119.5)		33.0 (4.3–135.0)		< 0.001
Symptoms occurrence in last week [*n* (%)]																		
*nd*		878		475		403				228		247		207		196		
Cough		274 (31)		203 (43)		71 (18)		< 0.001		137 (60)		66 (27)		30 (14)		41 (21)		< 0.001
Wheeze		92 (10)		41 (9)		50 (12)		0.067		24 (11)		17 (7)		11 (5)		39 (20)		< 0.001
Difficulty breathing		105 (12)		74 (16)		31 (8)		< 0.001		34 (15)		40 (16)		3 (1)		28 (14)		< 0.001
Cold symptoms		155 (18)		115 (24)		40 (10)		< 0.001		73 (32)		42 (17)		26 (13)		14 (7)		< 0.001
Missed school		90 (10)		79 (17)		11 (3)		< 0.001		38 (17)		41 (17)		5 (2)		6 (3)		< 0.001
SABA use		125 (14)		50 (11)		75 (19)		< 0.001		23 (10)		27 (11)		19 (9)		56 (29)		< 0.001
**a***p*-Values for *t*-tests or analyses of variance for continuous variables and chi-square tests (when all cell values > 5) or Fisher’s exact test for categorical variables. **b**Missing information for BMI (two subjects) and for caretaker education (eight subjects: five at EP-A and one at each of the other schools). **c**BMI for age and sex percentile values (Centers for Disease Control and Prevention 2011) assigned to BMI categories: normal, 5th–85th percentile; overweight, 85th–95th percentile; obese, ≥ 95th percentile. **d**Number of weekly eNO samples or symptoms diaries completed over the study period.																		

*eNO and symptoms data.* A total of 787 eNO samples were collected over the study period, with an average of 14 (range, 6–16) repeated measures per subject. Overall median eNO levels were 20.0 ppb (range, 2.5–135.0) (Table 1) and similar to those found in other panels of asthmatic children (Barraza-Villarreal et al. 2008; Delfino et al. 2006; Koenig et al. 2003; Liu et al. 2009), although we observed a wide variation in subject-specific median eNO levels (range, 6.8–89.3 ppb). Eleven children reported ETS exposure several times per month or more; median eNO levels remained the same for the total population when excluding these subjects from analyses (Table 1). A total of 878 diaries (each recording 7 days of data) were collected over the study period. Overall, the weekly reporting of cough, difficulty breathing, cold symptoms, and missed school was greater in CJ than in EP, and SABA (rescue inhaler) use was greater in EP than in CJ. These trends, together with ICS medication use patterns, may be partly related to differences in socioeconomic status and health care access across the cities.

*Air quality data.* Table 2 presents descriptive statistics for the exposure metrics of *a priori* interest in the epidemiologic analyses [i.e., 48-hr (Wednesday–Friday) PM and O_3_ concentrations and 96-hr (Monday–Friday) NO_2_ concentrations] for all sampling sites. Full results describing the air quality data set collected are provided elsewhere (Raysoni et al. 2011). Pollutant concentrations varied spatially across the outdoor school and CAMS sites, with higher mean values and standard deviations observed for all pollutants (except O_3_) in CJ than in EP. Among the outdoor school sites, PM concentrations at CJ-A (light traffic zone) were generally higher than at CJ-B (heavy traffic zone); however, exposure metrics specifically related to traffic (e.g., BC and NO_2_) were higher at CJ-B. In EP, concentrations at EP-B were generally higher than those at EP-A, as expected. The differences among indoor school concentrations mirrored those measured outdoors. For example, higher concentrations were observed in CJ schools than in EP schools. It is worth noting that NO_2_ levels indoors were higher than outdoors in CJ, particularly at CJ-B, which may be attributed to indoor combustion sources (gas heaters and indoor cooking activities near the sampler in CJ-B).

**Table 2:** Air pollution summary statistics.*^a^*

Indoor*b*							Outdoor*b*							Ambient*c*								
Statistic	CJ-A	CJ-B	EP-A	EP-B	CJ-A	CJ-B	EP-A	EP-B	CAMS 12	CAMS 37	CAMS 41	CAMS 49	CAMS 72
48-hr PM_10_ (μg/m^3^)																										
Mean		68.2		47.1		17.2		22.1		87.7		54.8		18.8		41.0		35.9				35.0		45.5		
SD		37.1		17.9		8.0		21.2		30.3		25.3		11.0		22.3		28.0				24.6		29.7		
Median		63.2		44.9		15.9		14.6		80.4		51.2		16.3		39.8		27.3				29.2		41.7		
IQR		45.4		24.4		8.9		14.8		41.1		36.1		13.8		19.1		10.1				11.5		20.6		
48-hr PM_10–2.5_ (μg/m^3^)																										
Mean		41.5		26.9		9.2		11.9		56.6		34.4		10.0		25.4										
SD		22.0		11.4		5.2		13.5		19.4		16.1		6.5		13.9										
Median		38.9		22.2		8.0		6.5		47.7		27.4		8.4		24.5										
IQR		15.5		13.8		3.9		7.4		33.8		21.7		8.0		13.7										
48-hr PM_2.5_ (μg/m^3^)																										
Mean		26.7		20.2		7.6		10.2		31.1		20.4		8.8		15.6		9.0				9.6				
SD		16.2		8.5		3.6		8.0		14.0		9.9		5.0		9.5		4.5				4.5				
Median		22.8		19.2		7		9.1		29.9		17.2		7.4		14.2		8.0				8.7				
IQR		19.0		11.9		6.7		6.9		25.7		11.9		6.5		6.6		3.0				4.9				
48-hr BC (μg/m^3^)																										
Mean		1.5		1.9		0.1		0.5		1.6		1.9		0.2		0.7										
SD		1.2		1.2		0.2		0.4		0.9		1.1		0.2		0.4										
Median		1.1		1.1		0.0		0.4		1.3		1.4		0.1		0.6										
IQR		2.4		1.9		0.2		0.4		1.7		1.8		0.4		0.5										
96-hr NO_2_ (ppb)																										
Mean		23.1		120.8		4.0		8.1		18.7		27.2		4.5		14.2		18.5		20.6		14.0				
SD		12.8		99.4		2.2		1.1		5.8		10.5		3.5		3.2		5.3		4.6		6.2				
Median		21.3		83.9		3.5		7.9		17.5		23.0		3.6		14.0		17.7		18.9		12.1				
IQR		14.6		182.3		2.3		1.1		4.6		17.4		3.6		4.4		5.5		6.7		9.6				
48-hr O_3_ (ppb)																										
Mean																		29.3		25.4		30.0		27.2		39.0
SD																		9.4		8.9		10.3		9.8		11.1
Median																		31.3		25.0		31.6		26.7		41.1
IQR																		11.9		9.9		13.2		10.5		18.1
Abbreviations: CAMS, continuous air monitoring station; IQR, interquartile range. **a**Only measurements ending on Fridays are included (e.g., 48-hr averages include Wednesday–Friday, and 96-hr averages include Monday–Friday). **b***n* = 15–16 for indoor and outdoor measurements. **c***n* = 17–18 for CAMS measurements.																										

Spearman correlations among the outdoor and indoor school metrics are presented in Supplemental Material, Table 1 (http://dx.doi.org/10.1289/ehp.1003169). Correlations between indoor and outdoor concentrations of the same pollutant varied considerably by school but were generally strongest for BC (*r* > 0.64) and PM_2.5_ (*r* > 0.53) and weaker for PM_10–2.5_ (*r* > 0.41) and NO_2_ (*r* > 0.01). This pattern is consistent with PM infiltration studies that indicate greater infiltration for smaller than for larger PM in the region (Li et al. 2003).

Within the indoor and outdoor school microenvironments, correlations among PM_10_, PM_10–2.5_, and PM_2.5_ at each school were strong [*r* ≥ 0.80 for most relationships, except with PM_10–2.5_ in several cases; Supplemental Material, Table 1 (http://dx.doi.org/10.1289/ehp.1003169)]. Correlations of BC and NO_2_ with size-fractionated PM were typically weaker at each school (*r* < 0.50 for most relationships), possibility indicating different temporal emission patterns of these traffic-related pollutants compared with the broader PM measures.

Correlations among outdoor school pollutant levels also varied, indicating spatiotemporal variability among the pollutants across the study sites [e.g., *r* = 0.36–0.93 for PM_2.5_; Supplemental Material, Table 1 (http://dx.doi.org/10.1289/ehp.1003169)]. Between each outdoor school site and CAMS 41, we observed correlations of 0.66–0.87 for PM_10_, 0.73–0.86 for PM_2.5_, and 0.63–0.90 for NO_2_. Correlations among the CAMS sites were higher: *r* = 0.76–0.87 for PM_10_, *r* = 0.91 for PM_2.5_, *r* = 0.90–0.91 for NO_2_, and *r* = 0.88–0.98 for O_3_.

*Epidemiologic associations.* All epidemiologic models predicting the occurrence of respiratory symptoms were consistent with the null (data not shown). In contrast, we observed positive overall associations between eNO and many of the measured pollutant metrics, with estimated increases in eNO from 1% to 3% per IQR increase in pollutant concentrations (Table 3). Exceptions were for outdoor BC and ambient (CAMS 41) PM_10_ and NO_2_, for which associations with eNO were weak and consistent with the null. Associations were similar when 11 ETS-exposed subjects were excluded [Supplemental Material, Table 2 (http://dx.doi.org/10.1289/ehp.1003169)].

**Table 3:** Associations between eNO and microenvironmental pollutant concentrations.*^a^*

Pollutant metric	IQR*b*	*nc*	Percent change in eNO (95% CI) per IQR increase	Chi-square	*p*-Value
48-hr PM_10_										
Ambient (CAMS 41)		11.5		733		0.1 (–0.3, 0.5)		0.33		0.568
Outdoor school		46.0		733		2.3 (0.7, 3.8)		8.19		0.004
Indoor school		41.1		733		3.2 (1.6, 4.8)		15.41		< 0.001
48-hr PM_10–2.5_										
Outdoor school		31.1		733		2.0 (0.3, 3.6)		5.59		0.018
Indoor school		25.3		733		2.8 (1.2, 4.5)		11.11		0.001
48-hr PM_2.5_										
Ambient (CAMS 41)		4.9		733		2.4 (1.3, 3.6)		16.80		< 0.001
Outdoor school		15.4		733		2.3 (1.0, 3.6)		12.00		0.001
Indoor school		14.5		733		2.7 (1.4, 3.9)		18.03		< 0.001
48-hr BC										
Outdoor school		1.0		733		0.3 (–0.8, 1.5)		0.30		0.584
Indoor school		1.1		733		1.4 (0.2, 2.7)		4.95		0.026
48-hr NO_2_										
Ambient (CAMS 41)		10.3		733		0.0 (–1.5, 1.4)		0.00		0.957
96-hr NO_2_										
Ambient (CAMS 41)		9.6		697		0.8 (–0.5, 2.1)		1.59		0.207
Outdoor school		12.3		697		3.8 (1.5, 6.1)		10.39		0.001
Indoor school		19.0		697		0.5 (0.1, 1.0)		6.08		0.014
48-hr O_3_										
Ambient (CAMS 41)		13.2		733		–0.2 (–1.9, 1.5)		0.05		0.825
**a**General linear mixed models with random subject effect and first-order autoregressive heterogeneous covariance structure and adjusted for school, indoor NO, ambient temperature, and relative humidity. **b**IQRs are in micrograms per cubic meter for PM_10_, PM_10–2.5_, PM_2.5_, and BC and in parts per billion for NO_2_ and O_3_; IQRs for outdoor and indoor school are from subject-specific assigned measurements and so are roughly equivalent to average IQRs across the four schools. **c**Analyses matched for missing data within the 48-hr and 96-hr pollutant metrics.										

Table 3 results allow for an overall comparison of the various exposure metrics. For PM measures, effect estimates from models using indoor school concentrations were slightly stronger and had lower *p*-values than corresponding models using outdoor school and ambient concentrations. In two-pollutant models including indoor and outdoor measures simultaneously, only associations of eNO with indoor metrics remained for PM_10–2.5_ and PM_2.5_ (two-pollutant models for BC were unstable; data not shown). In single-pollutant models (Table 3), the association with eNO was weaker for indoor NO_2_ than for outdoor NO_2_, but in a two-pollutant model, neither indoor nor outdoor NO_2_ measures were associated with eNO (data not shown). Models using ambient metrics generally showed the weakest associations, particularly for PM_10_ and NO_2_. Overall, among the pollutants, the estimated effects of PM_2.5_ were most similar among microenvironments: indoor school, 2.7% [95% confidence interval (CI): 1.4, 3.9]; outdoor school, 2.3% (95% CI: 1.0, 3.6); ambient, 2.4% (95% CI: 1.3, 3.6).

**Figure 2:**
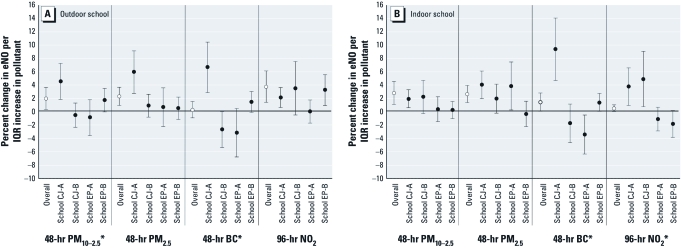
Overall and cohort-specific associations between eNO and outdoor (*A*) and indoor (*B*) pollutant metrics: results of general linear mixed models with random subject effect and first-order autoregressive heterogeneous covariance structure and adjusted for school, indoor NO, ambient temperature, and relative humidity, and the two-way interaction between pollution and school for cohort-specific associations. Overall associations were standardized using average IQRs across the schools (Table 3); cohort-specific associations were standardized using school-specific IQRs (Table 2). Models were matched for missing pollutant values by averaging time such that *n* = 733 for all 48-hr metrics and *n* = 697 for 96-hr NO_2_. Error bars reflect 95% CIs. Open circles, associations for full study population; solid circles, association for each school-based cohort. *p-Value for interaction < 0.05.

Because of the differences in characteristics across the four school-based cohorts, we computed cohort-specific results for each eNO–pollutant association [Figure 2; see Supplemental Material, Tables 3 and 4 (http://dx.doi.org/10.1289/ehp.1003169)]. We observed consistent positive associations with eNO and all pollutants (using both indoor and outdoor metrics) for cohort CJ-A. For CJ-A, indoor and outdoor BC had among the strongest associations, followed by outdoor PM_10–2.5_ and outdoor PM_2.5_. For the other cohorts, although associations were generally consistent with the null, for CJ-B there was indication of stronger associations of eNO with indoor and outdoor NO_2_ than with the other pollutants; for EP-B, with indoor and outdoor BC and outdoor NO_2_; and for EP-A, with indoor PM_2.5_.

Sensitivity analysis results are provided in the Supplemental Material and described briefly here. Generally, eNO–pollutant associations for the PM measures were robust to alternate temporal averaging times [i.e., 24, 48, 72, and 96 hr; see Supplemental Material, Figure 1 (http://dx.doi.org/10.1289/ehp.1003169)] and to alternative specifications of meteorologic control (data not shown). Associations for NO_2_ and O_3_ were not as consistent as those for PM_10_ and PM_2.5_ when comparing alternate temporal pollutant averages (see Supplemental Material, Figure 1) and were highly sensitive to model specification with respect to meteorology. For example, 96-hr outdoor school NO_2_ showed no association with eNO when including 24-, 48-, or 72-hr meteorologic variables but did show a significant association when using 96-hr meteorologic control [as conducted in our *a priori* analyses (Table 3); data not shown]. Finally, inclusion of 72-hr ambient O_3_ (the O_3_ averaging time most highly associated with eNO) in two-pollutant models with outdoor school 48-hr PM and 96-hr NO_2_ measures had no considerable influence on the PM and NO_2_ results (see Supplemental Material, Table 5). The only association for which the interpretation changed upon inclusion of O_3_ was that for BC, which was consistent with the null (–0.02%; 95% CI: –1.4%, 1.3%) in the single-pollutant model and positive (2.4%; 95% CI: 0.6%, 4.1%) in the two-pollutant model.

We assessed several subject-specific factors (sex, BMI category, hay fever status, ICS use, and caretaker education level) for their potential to modify the observed associations [Supplemental Material, Table 6 (http://dx.doi.org/10.1289/ehp.1003169)]. For BMI category, associations were consistently stronger in overweight and obese children (e.g., for PM_10–2.5_, 3.5%; 95% CI: 1.2%, 5.8%) than in normal-weight children (e.g., for PM_10–2.5_, 0.2%; 95% CI: –2.0%, 2.4%; *p*-value for interaction = 0.035; see Supplemental Material, Table 6, Figure 2). These patterns of association largely remained when stratifying the analyses by city.

## Discussion

Here, we present results of the first parallel binational study of the health impacts of air pollution along the U.S.–Mexico border, assessing concurrent measurements of air pollutant parameters in different microenvironments and children’s respiratory responses in CJ and EP. Two main goals of this study were to examine whether school-based monitors are more effective than ambient monitors for research and guiding policy, and which measures of traffic pollution are the most sensitive predictors of changes in the children’s health. This study also provided a unique opportunity to assess air pollution health effects across broad exposure and socioeconomic gradients, factors that may modify observed epidemiologic associations. Collectively, we observed positive, significant associations between eNO and measures of size-fractionated PM and BC, with findings of 1–3% increases in eNO per IQR increase in 48-hr pollutant concentrations. School-based models indicated consistent significant associations for the CJ-A cohort and less consistent associations for the other cohorts among the various pollutant metrics. These results may attest to a number of factors, including baseline differences in modifying risk factors among the cohorts, as well as limited power to detect subtle changes in acute inflammation in a cohort-specific modeling setting. Together, these results support previous findings showing associations between short-term exposure to air pollution and acute respiratory response in asthmatic children in the PdN region (Hernandez-Cadena et al. 2000; Holguin et al. 2007).

A central finding from the present analysis was that school-based pollutant measurements generally provided the strongest measures of effect. The results suggest that exposure misclassification, which can lead to attenuation of effects, may be greater when using ambient data than when using school-based pollutant metrics. This finding is consistent with other studies that have found more robust associations using personal exposures than using ambient data (Allen et al. 2008; Delfino et al. 2006). Indeed, it is likely that small-scale intraurban variability in pollutant concentrations, characteristic of traffic pollutants in particular, are generally not captured by measurements made at ambient monitoring sites. In the present study, we observed considerable spatial contrasts in air pollution levels across the school sites that were striking considering the shared airshed but consistent with results of previous monitoring campaigns in this area (Li et al. 2001; Noble et al. 2003). Li et al. (2001) observed that the high number of unpaved roads and brick kiln, automobile, and industrial emissions in CJ may be responsible for the higher fine and coarse PM concentrations in CJ than in EP. Within EP, Gonzales et al. (2005) reported concentration gradients ranging from 11.0 to 37.5 ppb for 7-day mean NO_2_ levels across 24 sites. The authors reported a decrement in NO_2_ concentrations with increasing distance from major roadways and international border crossings. These results are similar to those observed in the present study, with lower concentrations measured at school EP-A than at school EP-B (located in the lower valley, close to the border highway). Correlations among the CAMS sites, which are usually sited in areas with typical background concentrations, were higher than correlations among the outdoor school sites. Diurnal trends of hourly CAMS 41 data (data not shown) indicated strong morning and evening rush hour peak concentrations for NO_2_, and to lesser extents for PM_10_ and PM_2.5_, similar to previous results of studies (Noble et al. 2003). Although peak concentrations usually occurred when children were not in school (e.g., peak median NO_2_ concentrations occurred at 0600 hours and 2000 hours), the school-based monitors were closer to most subject’s homes than were the ambient monitors, and children were likely to be commuting to or from school, or to be outdoors near school, during rush-hour periods.

The PdN region is an area heavily affected by traffic emissions from one of the busiest U.S.–Mexico border crossings, as well as high local vehicle use. Although CJ has about four times the population density of EP, the estimated number of vehicle miles traveled per day in EP (15.8 × 10^6^; El Paso Metropolitan Planning Organization 2007) is almost four times the estimated number for CJ (4.1 × 10^6^; Wolf et al. 2003). In this study, we hypothesized that adverse respiratory response in our cohort may be associated with a component of traffic emissions as measured by BC or NO_2_. Among the pollutants we measured, we found that PM_2.5_ was the best predictor of eNO in each of sampling microenvironments. This finding may be attributable partly to lower measurement error for PM_2.5_ than for the other pollutants measured (e.g., compared with BC, for which measurements required additional laboratory analyses). Overall associations between eNO and BC were strong, however, when controlling for O_3_ [see Supplemental Material, Table 5 (http://dx.doi.org/10.1289/ehp.1003169)], and cohort-specific associations, which varied among the schools and measured pollutants, also suggest that both traffic and nontraffic emissions influenced eNO in this study.

Our results suggest that PM pollutant levels predict acute respiratory responses better than do NO_2_ levels in this population. Sensitivity analyses showed consistent and robust associations between PM and eNO, regardless of choice of pollutant averaging time, meteorologic control, or copollutant control. In contrast, associations between eNO and NO_2_ (and O_3_) were more sensitive to specification of pollutant averaging time and meteorologic control. Unstable results for the gaseous pollutants highlight the difficulty in determining appropriate methods for controlling for meteorology in air pollution epidemiologic models, particularly when analyzing covarying pollutants that are highly driven by meteorologic processes. Some of our NO_2_ measurements (particularly indoor NO_2_ at CJ schools) were also affected by indoor sources, a finding that clearly does not support the use of this pollutant as a traffic-pollutant metric for this particular cohort.

This study is the first to concurrently examine children in both the United States and Mexico, and the results provide initial insights into factors that may influence susceptibility and vulnerability to air pollution health effects across the PdN region. The large spatial contrasts in air pollution levels across the study area suggest large discrepancies in air pollution exposure levels across the cities and cohorts. These within- and between-city differences in exposure levels were partially matched by similar trends in observed epidemiologic associations, with generally stronger associations observed for CJ, particularly CJ-A, than for EP. Although environmental factors (e.g., increased magnitude and variability of exposure in CJ) may be an explanatory factor, our results may also reflect the influence of health and sociodemographic factors on pollutant–health associations. For example, we found significantly lower medication use, lower socioeconomic status, and higher symptom occurrence in CJ than in EP subjects, which may be attributable in part to differences in health care access. It is possible that better self-reporting by EP subjects on the questionnaires contributed to the observed differences in cohort characteristics. We also found that CJ subjects, particularly CJ-A subjects, had significantly higher BMI percentiles than did EP subjects. In analyses assessing BMI category as an effect modifier, we found significantly stronger associations in overweight and obese children than in normal-weight children. These patterns persisted when stratifying the analyses by city and indicate that BMI is a possible influential risk factor for adverse respiratory response associated with air pollution exposure for both sets of children. These findings are supported by previous research indicating obesity to be a risk factor for asthma (Gold and Wright 2005) and by initial studies of BMI/obesity, air pollution, and respiratory health (Alexeef et al. 2007; Bennett et al. 2007). Overall, the observed subject-specific differences by city and school suggest greater susceptibility and vulnerability of CJ (particularly CJ-A) subjects than of EP subjects, with BMI playing a possible role.

Although significant and robust to model specification, estimated effects of air pollution on eNO were small (e.g., 2.3% change in eNO per 15.4-μg/m^3^ increase in outdoor PM_2.5_). Although comparable to those found by some studies of asthmatic children (Barraza-Villarreal et al. 2008; Delfino et al. 2006; Liu et al. 2009), estimated effects were lower than those reported for an asthmatic children’s cohort in Seattle, for which changes in eNO (in children not using ICS) of approximately 20% were observed for 10 μg/m^3^ increases in PM_2.5_ (Koenig et al. 2003). Differences in estimated effects across studies may be attributable partly to differences in pollution composition and exposures across different regions, as well as differences in population susceptibility. Although we examined a similar at-risk population as Koenig et al. (2003), we did not directly ascertain asthma severity (e.g., via spirometry), which is likely to influence observed magnitudes of effect, and which limits our ability to fully interpret epidemiologic associations by school. The clinical significance of the estimated increases in eNO with pollutant levels observed in our study population is also unclear, particularly because corresponding changes in respiratory symptoms with the measured pollutant metrics were not observed. The largely null findings for associations with respiratory symptoms may be attributable to low power as well as measurement and recall error, which are common limitations of survey data for pediatric cohorts. Ultimately, our results suggest the presence of small subclinical changes in airway inflammation (as assessed by eNO) associated with air pollution exposures.

## Conclusions

This analysis presents results from the first binational study of the health impacts of air pollution along the U.S.–Mexico border. We found evidence of an acute adverse effect of air pollution on the subclinical respiratory health of asthmatic children in this region, with findings of positive associations between the measured pollutants and a measure of airway inflammation, but not respiratory symptoms. Traffic-related and non–traffic-related PM pollutants were more stable predictors of airway inflammation than was NO_2_. Observed associations differed significantly across the four school-based cohorts, consistent with heterogeneity in both pollutant concentrations and cohort characteristics. Finally, school-based pollutant measures were the best predictors of acute airway inflammation in these subjects. These results offer preliminary support for the use of air pollution monitors close to schools to track exposure and potential health risk in this susceptible population.

## Supplemental Material

(352 KB) PDF
