# Supplementary material for: Air Pollution and Acute Respiratory Response in a Panel of Asthmatic Children along the U.S.–Mexico Border
Source: Environ Health Perspect. 2011 Sep 6;120(3):437–44. doi: 10.1289/ehp.1003169 (PMC3295334; doi:10.1289/ehp.1003169)

SUPPLEMENTAL MATERIAL

AIR POLLUTION AND ACUTE RESPIRATORY RESPONSE IN A PANEL OF ASTHMATIC CHILDREN ALONG THE US-MEXICO BORDER

Stefanie Ebelt Sarnat[1], Amit U. Raysoni[2], Wen-Whai Li[2], Fernando Holguin[3], Brent Johnson[1], Silvia Flores-Luevano[2], Jose Humberto Garcia[2], Jeremy A. Sarnat[1]

[1]Emory University, Atlanta, GA
[2]The University of Texas at El Paso, El Paso, TX
[3]University of Pittsburgh Medical Center, Pittsburgh, PA

TABLE OF CONTENTS

Pg. 2 **Supplemental Material, Table 1.** Spearman's correlations among school-based indoor and outdoor pollutant concentrations (n=15-16).

Pg. 3 **Supplemental Material, Table 2.** Associations between eNO and microenvironmental pollutant concentrations for full cohort (58 subjects) and for non-environmental tobacco smoke exposed subset (47 subjects).

Pg. 4 **Supplemental Material, Table 3.** Overall and cohort-specific associations for outdoor school pollutant metrics (as presented in Figure 2.a in main text).

Pg. 5 **Supplemental Material, Table 4.** Overall and cohort-specific associations for indoor school pollutant metrics (as presented in Figure 2.b in main text).

Pg. 6 **Supplemental Material, Figure 1.** Associations between eNO and pollutant concentrations at different temporal averages: a) PM_{10}, b) $PM_{2.5}$, c) NO_2, d) O_3.

Pg. 7 **Supplemental Material, Table 5.** Comparison of single- and two-pollutant models with outdoor school PM and NO_2 and ambient O_3 measures.

Pg. 8 **Supplemental Material, Table 6.** Associations between eNO and outdoor school pollutant concentrations by subject-specific factors.

Pg. 10 **Supplemental Material, Figure 2.** Associations between eNO and outdoor school pollutant metrics by BMI category for the whole study population and by city.

Supplemental Material, Table 1. Spearman's correlations among school-based indoor and outdoor pollutant concentrations (n=15-16).[a]

Pollutant		48-hr PM_{10}				48-hr $PM_{10-2.5}$				48-hr $PM_{2.5}$				48-hr BC				96-hr NO_2			
	**School**	CJ-A	CJ-B	EP-A	EP-B	CJ-A	CJ-B	EP-A	EP-B	CJ-A	CJ-B	EP-A	EP-B	CJ-A	CJ-B	EP-A	EP-B	CJ-A	CJ-B	EP-A	EP-B
**48-hr PM_{10}**	CJ-A	0.60	0.45	0.60	0.82	0.97	0.34	0.48	0.56	0.94	0.51	0.68	0.81	0.16	-0.06	-0.01	-0.13	-0.50	-0.26	-0.28	-0.19
	CJ-B	0.78	0.66	0.48	0.50	0.38	0.93	0.41	0.30	0.49	0.88	0.40	0.59	-0.19	0.16	-0.09	0.17	-0.48	-0.34	-0.09	-0.40
	EP-A	0.68	0.28	0.75	0.85	0.60	0.55	0.86	0.87	0.45	0.27	0.84	0.73	-0.36	-0.47	-0.44	-0.49	-0.78	-0.71	-0.19	0.25
	EP-B	0.86	0.69	0.80	0.76	0.78	0.44	0.81	0.87	0.73	0.41	0.78	0.94	-0.30	-0.31	-0.35	-0.44	-0.73	-0.64	-0.40	0.12
**48-hr $PM_{10-2.5}$**	CJ-A	0.84	0.62	0.67	0.88	0.41	0.27	0.52	0.58	0.84	0.40	0.60	0.76	0.06	-0.21	-0.09	-0.20	-0.58	-0.25	-0.33	-0.10
	CJ-B	0.71	0.97	0.28	0.68	0.65	0.49	0.43	0.39	0.34	0.68	0.45	0.48	-0.32	0.00	-0.16	0.05	-0.56	-0.50	-0.16	-0.26
	EP-A	0.69	0.28	0.97	0.81	0.73	0.29	0.90	0.92	0.31	0.26	0.54	0.65	-0.58	-0.46	-0.48	-0.38	-0.85	-0.70	-0.26	0.27
	EP-B	0.78	0.61	0.79	0.93	0.93	0.66	0.81	0.60	0.40	0.09	0.71	0.70	-0.55	-0.58	-0.49	-0.56	-0.83	-0.72	-0.40	0.28
**48-hr $PM_{2.5}$**	CJ-A	0.86	0.75	0.47	0.63	0.53	0.65	0.41	0.48	0.84	0.60	0.64	0.78	0.35	0.16	0.12	-0.06	-0.29	-0.13	-0.17	-0.31
	CJ-B	0.87	0.91	0.37	0.66	0.61	0.83	0.37	0.55	0.93	0.91	0.22	0.50	0.14	0.44	0.15	0.38	-0.23	-0.04	0.13	-0.64
	EP-A	0.63	0.23	0.96	0.71	0.53	0.21	0.88	0.66	0.52	0.36	0.53	0.74	-0.02	-0.29	-0.32	-0.54	-0.56	-0.54	-0.35	0.08
	EP-B	0.78	0.56	0.72	0.90	0.67	0.50	0.71	0.72	0.70	0.63	0.71	0.89	-0.20	-0.18	-0.31	-0.40	-0.62	-0.53	-0.43	0.06
**48-hr BC**	CJ-A	0.08	0.43	-0.54	-0.16	-0.10	0.35	-0.51	-0.24	0.23	0.43	-0.51	-0.19	0.83	0.68	0.63	0.37	0.66	0.61	0.30	-0.43
	CJ-B	0.26	0.54	-0.34	-0.03	0.06	0.50	-0.33	-0.06	0.36	0.53	-0.31	-0.07	0.85	0.95	0.76	0.83	0.54	0.45	0.51	-0.44
	EP-A	0.45	0.57	-0.29	-0.02	0.33	0.55	-0.28	0.06	0.53	0.63	-0.36	-0.10	0.58	0.71	0.64	0.71	0.51	0.42	0.58	-0.25
	EP-B	0.24	0.52	-0.29	-0.07	0.22	0.57	-0.29	0.03	0.18	0.40	-0.32	-0.24	0.65	0.86	0.69	0.93	0.34	0.32	0.56	-0.37
**96-hr NO_2**	CJ-A	0.11	0.14	-0.04	0.12	0.34	0.24	0.08	0.34	0.00	0.18	-0.20	-0.06	0.26	0.30	0.54	0.43	0.36	0.75	0.51	-0.25
	CJ-B	-0.21	0.01	-0.60	-0.41	-0.28	0.02	-0.53	-0.34	-0.13	0.05	-0.66	-0.48	0.58	0.57	0.51	0.52	0.50	0.92	0.47	-0.51
	EP-A	0.21	0.54	-0.30	0.00	0.21	0.62	-0.24	0.17	0.21	0.40	-0.39	-0.16	0.59	0.74	0.73	0.84	0.67	0.59	0.66	-0.16
	EP-B	-0.04	0.16	-0.34	-0.10	0.14	0.24	-0.26	0.04	-0.20	0.05	-0.44	-0.28	0.41	0.47	0.41	0.68	0.74	0.57	0.67	0.01

[a]Averaging periods are 48-hr for PM and 96-hr for NO_2, with all averaging periods ending on Fridays (e.g., 48-hr averages include Wed-Fri period, 96-hr averages include Mon-Fri period); bottom half of table (non-shaded cells) present outdoor-outdoor correlations; top half of table (light shaded cells) present indoor-indoor correlations; middle diagonal (dark shaded cells) present indoor-outdoor correlations.

Supplemental Material, Table 2. Associations between eNO and microenvironmental pollutant concentrations for full cohort (58 subjects) and for non-environmental tobacco smoke exposed subset (47 subjects).[a]

			Full Cohort (58 subjects)				Non-ETS Exposed Cohort (47 subjects)			
Pollutant	Pollutant Metric	IQR[b]	N[c]	% Change in eNO (95% CI) per IQR increase	χ^2	P-value	N[c]	% Change in eNO (95% CI) per IQR increase	χ^2	P-value
48-hr PM_{10}	Ambient (CAMS 41)	11.5	733	0.1 (-0.3-0.5)	0.33	0.568	593	0.2 (-0.3-0.6)	0.60	0.439
	School Outdoor	46.0	733	2.3 (0.7-3.8)	8.19	0.004	593	2.3 (0.6-4.0)	6.83	0.009
	School Indoor	41.1	733	3.2 (1.6-4.8)	15.41	<0.001	593	3.7 (1.9-5.5)	16.05	<0.001
48-hr $PM_{10-2.5}$	School Outdoor	31.1	733	2.0 (0.3-3.6)	5.59	0.018	593	1.9 (0.1-3.8)	4.19	0.041
	School Indoor	25.3	733	2.8 (1.2-4.5)	11.11	0.001	593	3.1 (1.2-5.0)	10.51	0.001
48-hr $PM_{2.5}$	Ambient (CAMS41)	4.9	733	2.4 (1.3-3.6)	16.80	<0.001	593	2.6 (1.3-3.9)	14.99	<0.001
	School Outdoor	15.4	733	2.3 (1.0-3.6)	12.00	0.001	593	2.4 (1.0-3.9)	11.32	0.001
	School Indoor	14.5	733	2.7 (1.4-3.9)	18.03	<0.001	593	3.1 (1.7-4.4)	19.76	<0.001
48-hr BC	School Outdoor	1.0	733	0.3 (-0.8-1.5)	0.30	0.584	593	0.3 (-1.0-1.6)	0.22	0.637
	School Indoor	1.1	733	1.4 (0.2-2.7)	4.95	0.026	593	1.4 (-0.1-2.8)	3.57	0.059
96-hr NO_2	Ambient (CAMS41)	9.6	697	0.8 (-0.5-2.1)	1.59	0.207	563	0.1 (-1.4-1.6)	0.02	0.900
	School Outdoor	12.3	697	3.8 (1.5-6.1)	10.39	0.001	563	3.6 (1.1-6.2)	8.07	0.005
	School Indoor	19.0	697	0.5 (0.1-1.0)	6.08	0.014	563	0.6 (0.2-1.1)	8.26	0.004

Abbreviations: IQR=interquartile range.

[a]General linear mixed models with random subject effect, first order autoregressive heterogeneous covariance structure, and adjusted for school, indoor NO, ambient temperature and relative humidity.

[b]IQRs in $\mu g/m^3$ for PM_{10}, $PM_{10-2.5}$, $PM_{2.5}$, and BC; and in ppb for NO_2; IQRs for Outdoor and Indoor School from subject-specific assigned measurements, thus roughly equivalent to average IQRs across the four schools.

[c]Analyses matched for missing data within the 48-hr and 96-hr pollutant metrics.

Supplemental Material, Table 3. Overall and cohort-specific associations for outdoor school pollutant metrics (as presented in Figure 2.a in main text).[a]

Pollutant	Cohort	IQR[b]	% Change in eNO (95% CI) per IQR increase	P-value	Intx P-value[c]
48-hr $PM_{10-2.5}$	Overall	31.1	2.0 (0.3-3.6)	0.018	n/a
	School CJ-A	33.8	4.5 (1.8-7.2)	0.001	0.024
	School CJ-B	21.7	-0.5 (-2.3-1.3)	0.577	
	School EP-A	8.0	-0.9 (-3.5-1.8)	0.524	
	School EP-B	13.7	1.7 (0.0-3.5)	0.048	
48-hr $PM_{2.5}$	Overall	15.4	2.3 (1.0-3.6)	0.001	n/a
	School CJ-A	25.7	6.0 (2.8-9.1)	<0.001	0.370
	School CJ-B	11.9	0.9 (-0.7-2.6)	0.268	
	School EP-A	6.5	0.7 (-2.1-3.5)	0.625	
	School EP-B	6.6	0.5 (-1.1-2.1)	0.524	
48-hr BC	Overall	1.0	0.3 (-0.8-1.5)	0.584	n/a
	School CJ-A	1.7	6.7 (2.9-10.4)	<0.001	<0.001
	School CJ-B	1.8	-2.7 (-5.3--0.1)	0.044	
	School EP-A	0.4	-3.2 (-6.7-0.4)	0.083	
	School EP-B	0.5	1.5 (0.0-3.0)	0.055	
96-hr NO_2	Overall	12.3	3.8 (1.5-6.1)	0.001	n/a
	School CJ-A	4.6	2.1 (0.7-3.6)	0.003	0.078
	School CJ-B	17.4	3.5 (-0.4-7.5)	0.080	
	School EP-A	3.6	0.0 (-1.7-1.7)	0.978	
	School EP-B	4.4	3.2 (1.0-5.5)	0.005	

Abbreviations: IQR=interquartile range; Intx=interaction.

[a]General linear mixed models with random subject effect, first order autoregressive heterogeneous covariance structure, and adjusted for school, indoor NO, ambient temperature and relative humidity, and the two-way interaction between pollution and school for cohort-specific associations. Analyses matched for missing data within the 48-hr and 96-hr pollutant metrics, such that N=733 for $PM_{10-2.5}$, $PM_{2.5}$ and BC models and N=697 for NO_2 models.

[b]IQRs in $\mu g/m^3$ for PM_{10}, $PM_{10-2.5}$, $PM_{2.5}$, and BC; and in ppb for NO_2; overall IQRs from subject-specific assigned measurements, thus roughly equivalent to average IQRs across the four schools.

[c]P-value from product term of pollutant*school.

Supplemental Material, Table 4. Overall and cohort-specific associations for indoor school pollutant metrics (as presented in Figure 2.b in main text).[a]

Pollutant	Cohort	IQR[b]	% Change in eNO (95% CI) per IQR increase	P-value	Intx P-value[c]
48-hr $PM_{10-2.5}$	Overall	25.3	2.8 (1.2-4.5)	0.001	n/a
	School CJ-A	15.5	1.9 (0.7-3.2)	0.003	0.754
	School CJ-B	13.8	2.3 (-0.1-4.6)	0.064	
	School EP-A	3.9	0.4 (-1.4-2.2)	0.647	
	School EP-B	7.4	0.3 (-0.9-1.5)	0.631	
48-hr $PM_{2.5}$	Overall	14.5	2.7 (1.4-3.9)	<0.001	n/a
	School CJ-A	19.0	4.1 (2.1-6.0)	<0.001	0.154
	School CJ-B	11.9	2.0 (-0.1-4.1)	0.061	
	School EP-A	6.7	3.9 (0.3-7.4)	0.032	
	School EP-B	6.9	-0.3 (-2.1-1.5)	0.745	
48-hr BC	Overall	1.1	1.4 (0.2-2.7)	0.026	n/a
	School CJ-A	2.4	9.4 (4.8-14.0)	<0.001	<0.001
	School CJ-B	1.9	-1.7 (-4.5-1.1)	0.232	
	School EP-A	0.2	-3.4 (-6.3--0.6)	0.019	
	School EP-B	0.4	1.4 (0.1-2.7)	0.030	
96-hr NO_2	Overall	19.0	0.5 (0.1-1.0)	0.014	n/a
	School CJ-A	14.6	3.8 (1.1-6.5)	0.006	0.008
	School CJ-B	182.3	4.9 (0.9-9.0)	0.017	
	School EP-A	2.3	-1.1 (-2.8-0.6)	0.198	
	School EP-B	1.1	-1.8 (-3.7-0.1)	0.066	

Abbreviations: IQR=interquartile range; Intx=interaction.

[a]General linear mixed models with random subject effect, first order autoregressive heterogeneous covariance structure, and adjusted for school, indoor NO, ambient temperature and relative humidity, and the two-way interaction between pollution and school for cohort-specific associations. Analyses matched for missing data within the 48-hr and 96-hr pollutant metrics, such that N=733 for $PM_{10-2.5}$, $PM_{2.5}$ and BC models and N=697 for NO_2 models.

[b]IQRs in $\mu g/m^3$ for PM_{10}, $PM_{10-2.5}$, $PM_{2.5}$, and BC; and in ppb for NO_2; overall IQRs from subject-specific assigned measurements, thus roughly equivalent to average IQRs across the four schools.

[c]P-value from product term of pollutant*school.

Supplemental Material, Figure 1. Associations between eNO and pollutant concentrations at different temporal averages: a) PM_{10}, b) $PM_{2.5}$, c) NO_2, d) O_3. Results of general linear mixed models with random subject effect, first order autoregressive heterogeneous covariance structure, and adjusted for school, indoor NO, ambient temperature and relative humidity. Associations standardized using IQRs specific to each temporal average. Models matched for missing values within pollutant, such that N=547 for all PM_{10} models, N=575 for all $PM_{2.5}$ models, N=759 for all NO_2 models, and N=579 for all O_3 models. Error bars reflect 95% confidence intervals.

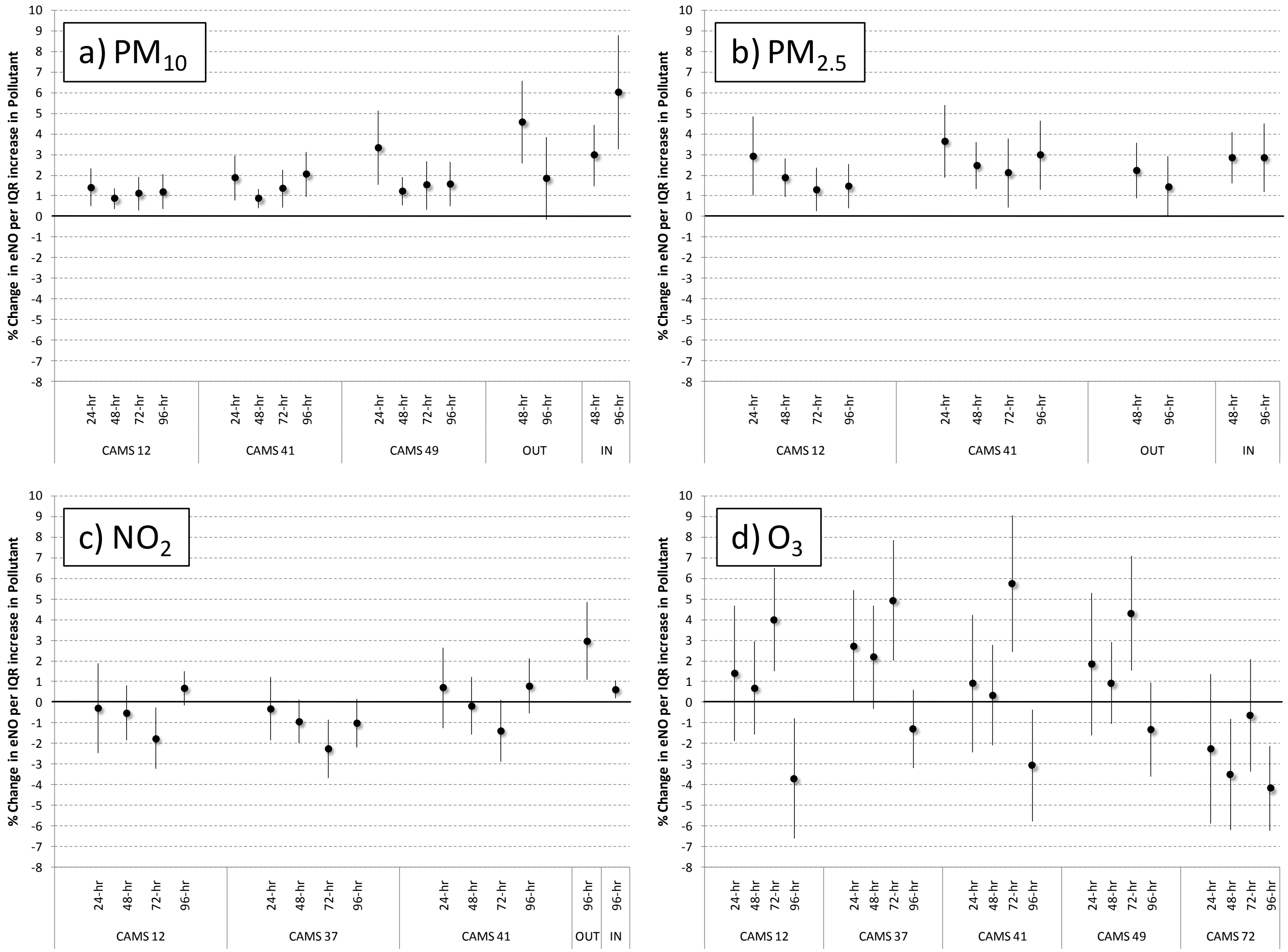

Supplemental Material, Table 5. Comparison of single- and two-pollutant models with outdoor school PM and NO_2 and ambient O_3 measures.[a]

			Outdoor School Parameter Estimates				72-hr O_3 Parameter Estimates			
Outdoor School Pollutant Metrics	Model	N	IQR[b]	% Change in eNO (95% CI) per IQR increase	χ^2	P-value	IQR[b]	% Change in eNO (95% CI) per IQR increase	χ^2	P-value
**48-hr PM_{10}**	1-pollutant[c]	719	46.0	1.5 (0.0-3.1)	4.01	0.046	13.0	2.9 (1.3-4.5)	12.26	<0.001
	2-pollutant[d]	719	46.0	2.2 (0.6- 3.8)	7.61	0.006	13.0	3.4 (1.7-5.0)	15.56	<.0001
**48-hr $PM_{10-2.5}$**	1-pollutant	719	27.9	1.1 (-0.3- 2.6)	2.28	0.131	13.0	2.9 (1.3-4.5)	12.26	<0.001
	2-pollutant	719	27.9	1.9 (0.3- 3.4)	5.68	0.017	13.0	3.4 (1.7-5.1)	15.56	<0.001
**48-hr $PM_{2.5}$**	1-pollutant	719	15.4	1.7 (0.4- 2.9)	6.89	0.009	13.0	2.9 (1.3-4.5)	12.26	<0.001
	2-pollutant	719	15.4	2.0 (0.7- 3.2)	9.44	0.002	13.0	3.2 (1.5-4.8)	14.17	<0.001
**48-hr BC**	1-pollutant	719	1.0	-0.02 (-1.4-1.3)	0.00	0.980	13.0	2.9 (1.3-4.5)	12.26	<0.001
	2-pollutant	719	1.0	2.4 (0.6- 4.1)	7.19	0.008	13.0	4.6 (2.5-6.6)	19.36	<0.001
**96-hr NO_2**	1-pollutant	705	10.2	3.7 (1.8- 5.7)	14.14	<0.001	13.0	-0.1 (-1.6-1.4)	0.03	0.873
	2-pollutant	705	10.2	4.4 (2.3- 6.6)	15.96	<0.001	13.0	1.0 (-0.5-2.6)	1.70	0.193

[a]General linear mixed models with random subject effect, first order autoregressive heterogeneous covariance structure, and adjusted for school, indoor NO, ambient temperature and relative humidity, and 72-hr O_3 in two-pollutant models.
[b]IQRs in µg/m^3 for PM_{10}, $PM_{10-2.5}$, $PM_{2.5}$, and BC; and in ppb for NO_2 and O_3; IQRs from subject-specific assigned measurements, thus roughly equivalent to average IQRs across the four schools.
[c]1-pollutant model results are those from single-pollutant models of PM or NO_2 and for O_3 (right column).
[d]2-pollutant model results show the coefficients from PM or NO_2 and O_3 when included in models simultaneously.

Supplemental Material, Table 6. Associations between eNO and outdoor school pollutant concentrations by subject-specific factors.[a]

Pollutant (IQR)[b]	Factor	Subjects[c]	Level	% Change in eNO (95% CI) per IQR increase	P-value	Intx P-value[d]
**48-hr $PM_{10-2.5}$** (IQR=31.1 μg/m^3)	Gender	All	Female	2.4 (-0.1-4.9)	0.057	0.671
		All	Male	1.7 (-0.4-3.8)	0.110	
	BMI Category[e]	All	Normal	0.2 (-2.0-2.4)	0.841	0.035
		All	Overweight+	3.5 (1.2-5.8)	0.003	
		CJ	Normal	-0.9 (-3.5-1.7)	0.491	0.020
		CJ	Overweight+	3.1 (0.5-5.7)	0.021	
		EP	Normal	0.9 (-2.3-4.1)	0.567	0.935
		EP	Overweight+	0.7 (-3.2-4.6)	0.719	
	Hay Fever	All	No	1.5 (-0.5-3.4)	0.145	0.312
		All	Yes	3.1 (0.4-5.9)	0.026	
	ICS Use	All	No	1.9 (0.2-3.6)	0.026	0.628
		All	Yes	3.5 (-2.5-9.5)	0.257	
	Caretaker Education	All	<HS	1.9 (-0.4-4.3)	0.107	0.931
		All	≥HS	2.1 (-0.4-4.5)	0.096	
**48-hr $PM_{2.5}$** (IQR=15.4 μg/m^3)	Gender	All	Female	2.2 (0.3-4.2)	0.027	0.933
		All	Male	2.4 (0.7-4.0)	0.006	
	BMI Category[e]	All	Normal	1.2 (-0.6-2.9)	0.198	0.156
		All	Overweight+	3.0 (1.1-4.9)	0.002	
		CJ	Normal	1.2 (-0.9-3.3)	0.260	0.081
		CJ	Overweight+	3.7 (1.6-5.9)	0.001	
		EP	Normal	1.3 (-1.7-4.3)	0.394	0.542
		EP	Overweight+	-0.2 (-4.1-3.7)	0.915	
	Hay Fever	All	No	2.1 (0.5-3.7)	0.008	0.641
		All	Yes	2.7 (0.5-5.0)	0.018	
	ICS Use	All	No	2.1 (0.8-3.5)	0.002	0.414
		All	Yes	4.1 (-0.5-8.7)	0.079	
	Caretaker Education	All	<HS	2.2 (0.3-4.0)	0.020	0.709
		All	≥HS	2.7 (0.7-4.7)	0.010	

Supplemental Material, Table 6. Cont'd.

Pollutant (IQR)[b]	Factor	Subjects[c]	Level	% Change in eNO (95% CI) per IQR increase	P-value	Intx P-value[d]
**48-hr BC** (IQR=1.0 µg/m^3)	Gender	All	Female	1.1 (-0.7-2.9)	0.245	0.266
		All	Male	-0.2 (-1.7-1.2)	0.767	
	BMI Category[e]	All	Normal	-1.5 (-2.9-0.0)	0.045	0.001
		All	Overweight+	2.1 (0.4-3.7)	0.014	
		CJ	Normal	-0.9 (-2.3-0.6)	0.249	0.006
		CJ	Overweight+	2.1 (0.4-3.7)	0.017	
		EP	Normal	-0.2 (-4.4-4.0)	0.914	0.017
		EP	Overweight+	6.4 (1.7-11.2)	0.008	
	Hay Fever	All	No	-0.4 (-1.7-1.0)	0.583	0.058
		All	Yes	1.9 (-0.1-3.9)	0.060	
	ICS Use	All	No	0.5 (-0.7-1.7)	0.430	0.116
		All	Yes	-3.4 (-8.2-1.3)	0.158	
	Caretaker Education	All	<HS	1.0 (-0.6-2.6)	0.216	0.253
		All	≥HS	-0.4 (-2.1-1.4)	0.683	
**96-hr NO_2** (IQR=12.3 ppb)	Gender	All	Female	4.0 (0.7-7.3)	0.017	0.823
		All	Male	3.6 (0.9-6.2)	0.009	
	BMI Category[e]	All	Normal	2.9 (0.3-5.6)	0.030	0.385
		All	Overweight+	4.4 (1.4-7.4)	0.004	
		CJ	Normal	n/a[f]		
		CJ	Overweight+	n/a[f]		
		EP	Normal	3.4 (-1.6-8.3)	0.185	0.134
		EP	Overweight+	8.6 (2.0-15.1)	0.010	
	Hay Fever	All	No	3.6 (1.0-6.2)	0.007	0.835
		All	Yes	4.0 (0.6-7.5)	0.023	
	ICS Use	All	No	4.0 (1.6-6.3)	0.001	0.295
		All	Yes	0.7 (-5.6-6.9)	0.835	
	Caretaker Education	All	<HS	4.4 (1.5-7.3)	0.003	0.586
		All	≥HS	3.4 (0.3-6.4)	0.032	

Abbrev: IQR=interquartile range; CJ=Ciudad Juarez; EP=El Paso; ICS=inhaled corticosteroid user; LT=leukotriene blocker user; HS=high school education.
[a]General linear mixed models with random subject effect, first order autoregressive heterogeneous covariance structure, and adjusted for school, indoor NO, ambient temperature and relative humidity, the subject-specific factor of interest, and the two-way interaction between pollution and the subject-specific factor.
[b]IQRs from subject-specific assigned measurements, thus roughly equivalent to average IQRs across the four schools.
[c]Subjects included in analysis: all=58 subjects; CJ=29 Ciudad Juarez subjects only; EP=29 El Paso subjects only.
[d]P-value from product term of pollutant*factor.
[e]BMI categories: normal=5th-85th pctl; overweight+≥85th pctl (overweight or obese).
[f]Model did not converge.

Supplemental Material, Figure 2. Associations between eNO and outdoor school pollutant metrics by BMI category for the whole study population and by city. Results of general linear mixed models with random subject effect, first order autoregressive heterogeneous covariance structure, and adjusted for school, indoor NO, ambient temperature and relative humidity, BMI category, and the two-way interaction between pollution and BMI category. Associations standardized using average IQRs across the four schools (see Supplementary Material Table 6). Error bars reflect 95% confidence intervals. Abbreviations: N=Normal (open symbols); O=Overweight or Obese (closed symbols); Full=all subjects (circles); CJ=Ciudad Juarez subjects (triangles); EP=El Paso subjects (squares). *p-value for interaction <0.05.

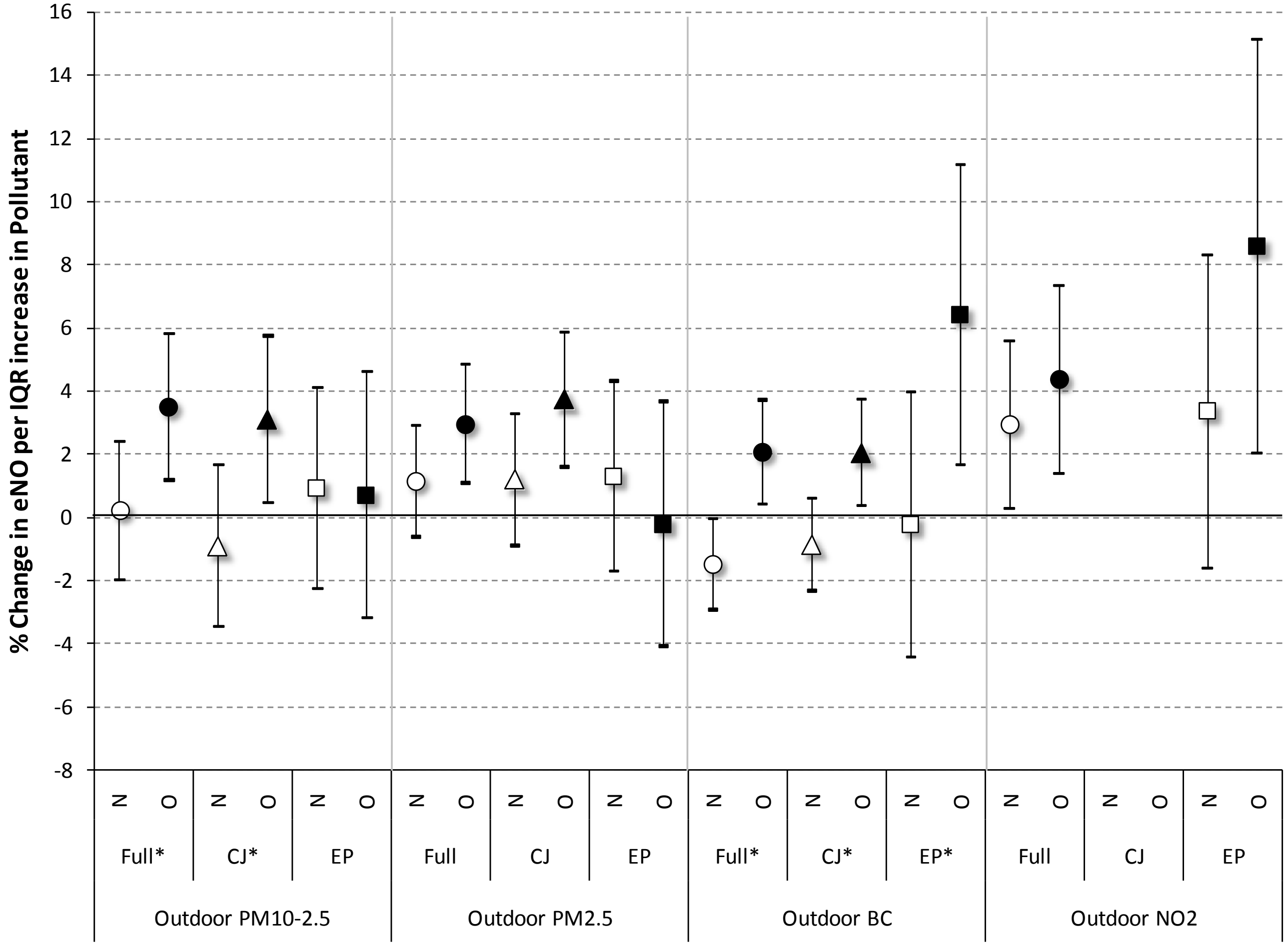

Supplement: (352 KB) PDF [file ehp.1003169.s001.pdf]
